# Antioxidant and Pro-Oxidant Activities of Melatonin in the Presence of Copper and Polyphenols In Vitro and In Vivo

**DOI:** 10.3390/cells8080903

**Published:** 2019-08-15

**Authors:** Jiajia Wang, Xiaoxiao Wang, Yufeng He, Lijie Jia, Chung S. Yang, Russel J. Reiter, Jinsong Zhang

**Affiliations:** 1 Laboratory of Redox Biology, State Key Laboratory of Tea Plant Biology and Utilization, School of Tea & Food Science, Anhui Agricultural University, Hefei 230000, China; 2 Department of Chemical Biology, Ernest Mario School of Pharmacy, Rutgers, The State University of New Jersey, Piscataway, NJ 08854, USA; 3 International Joint Laboratory on Tea Chemistry and Health Effects, Anhui Agricultural University, Hefei 230000, China; 4 Department of Cellular and Structural Biology, UT Health Science Center, San Antonio, TX 78229, USA

**Keywords:** melatonin, polyphenol, copper, ROS, DNA

## Abstract

Melatonin is a well-documented antioxidant. Physicochemical analysis using the density functional theory suggests that melatonin is a copper chelating agent; however, experimental evidence is still in demand. The present study investigated the influence of melatonin on reactive oxygen species (ROS) generated from polyphenol autoxidation in the presence of copper. Surprisingly, we found that melatonin paradoxically enhanced ROS formation in a redox system containing low concentrations of copper and quercetin (Que) or (−)-epigallocatechin-3-gallate (EGCG), due to reduction of cupric to cuprous ion by melatonin. Addition of DNA to this system inhibited ROS production, because DNA bound to copper and inhibited copper reduction by melatonin. When melatonin was added to a system containing high concentrations of copper and Que or EGCG, it diminished hydroxyl radical formation as expected. Upon addition of DNA to high concentrations of copper and Que, this pro-oxidative system generated ROS and caused DNA damage. The DNA damage was not prevented by typical scavengers of hydroxyl radical DMSO or mannitol. Under these conditions, melatonin or bathocuproine disulfonate (a copper chelator) protected the DNA from damage by chelating copper. When melatonin was administered intraperitoneally to mice, it inhibited hepatotoxicity and DNA damage evoked by EGCG plus diethyldithiocarbamate (a copper ionophore). Overall, the present study demonstrates the pro-oxidant and antioxidant activities of melatonin in the redox system of copper and polyphenols. The pro-oxidant effect is inhibited by the presence of DNA, which prevents copper reduction by melatonin. Interestingly, in-vivo melatonin protects against copper/polyphenol-induced DNA damage probably via acting as a copper-chelating agent rather than a hydroxyl radical scavenger. Melatonin with a dual function of scavenging hydroxyl radical and chelating copper is a more reliable DNA guardian than antioxidants that only have a single function of scavenging hydroxyl radical.

## 1. Introduction

Melatonin is secreted by the pineal gland and is important in the regulation of biorhythms. It is also found in many medicinal and food plants [1,2,3]. Evidence gathered to date strongly indicates that melatonin is a powerful free radical scavenger [1,2,3,4]. Thus, dietary melatonin supplementation via edible plants has been suggested to be a promising preventive or therapeutic approach for a variety of diseases [1,2,3]. Physicochemical analysis using the density functional theory suggests that melatonin is also a copper chelating agent [5]. The combination of Cu(II) and H_2_O_2_ constitutes a Fenton-like system producing hydroxyl radicals. Melatonin protected against the Fenton-like system evoked damage of bovine serum albumin via neutralizing hydroxyl radical [6]. Paradoxically, melatonin acts as an extremely strong pro-oxidant in the Fenton-like system probably because it chemically reduces copper [7]. Although the copper-reducing effect of melatonin has not been reported, serotonin (the precursor of melatonin) and 6-hydroxymelatonin (the metabolite of melatonin) have been documented to reduce copper [8,9,10]. The pro-oxidant action of melatonin is not limited in the Fenton-like system; melatonin promotes lateral root formation via increasing hydrogen peroxide levels [11] and kills cancer cells by promoting overt oxidative stress [12,13] or via increasing oxidative stress of electrophiles such as p-benzoquinone methide [14].

Over 8000 polyphenolic compounds are found in the human diet [15]. Tea, coffee, red wine, and many fruits and vegetables contain abundant polyphenols. Mounting evidence suggests that polyphenol consumption may prevent and alleviate chronic diseases [16,17]. However, adverse effects due to copious consumption of extracted polyphenols have been documented [16,17]. Green tea extracts with (-)-epigallocatechin-3-gallate (EGCG) being the predominant catechin have been marketed as a weight loss product. Hepatoxicity occurred in some individuals consuming the product [18,19,20]. Chlorogenic acid, a major polyphenol of coffee, has been reported to have many health-beneficial effects; however, it also causes cytotoxicity/genotoxicity [21]. In addition to well-known antioxidant functions, many dietary polyphenols possess pro-oxidant properties, by generating reactive oxygen species (ROS) [22,23]. The pro-oxidant effect of polyphenols is considered to be a principal mechanism for adverse responses, especially in the case of EGCG [24]. The pro-oxidant action of EGCG can be dramatically enhanced by copper. EGCG in combination with diethyldithiocarbamate (DEDTC) (metabolite of disulfiram, a drug for the treatment of alcoholism) induced severe liver injury in mice due to the DEDTC-mediated increase of hepatic copper [25]. A number of in-vitro studies have demonstrated that polyphenols and copper cooperatively cause DNA damage [26]. Such an action may account for the potential mutagenic effect of polyphenols [27,28] since copper is presented in the nucleus and is closely associated with chromosomes and DNA bases [29].

Both melatonin and polyphenols exist in medicinal and food plants, and both agents are recommended for supplementation [1,2,3]. Whether melatonin scavenges copper/polyphenol produced ROS and protects against copper/polyphenol evoked DNA damage remains unclear. This cannot be accurately extrapolated from melatonin’s capacity of directly scavenging ROS and/or chelating copper, because (1) melatonin acts as a potent pro-oxidant in the Fenton-like system of Cu(II)/H_2_O_2_ [7]; (2) many dietary polyphenols generate hydrogen peroxide via autoxidation [23]; and (3) hydrogen peroxide is an important mediator for copper/polyphenol evoked DNA damage [29]. Thus, the present study scrutinized the role of melatonin in the redox system of copper and dietary polyphenol in the absence or presence of DNA.

## 2. Materials and Methods

### 2.1. Chemicals and Drugs

EGCG was obtained from Ebeikar Tea & Extracts Co., Ltd. (Hangzhou, China). Melatonin, quercetin (Que), luteolin, chlorogenic acid, DEDTC, 2′, 7′-dichlorofluorescin diacetate (DCFH-DA), coumarin-3-carboxylic acid (3-CCA), bathocuproinedisulfonic acid disodium salt (BCS), neocuproine, catalase, mannitol, superoxide dismutase (SOD), histidine, and methionine were purchased from Sigma (St. Louis, Missouri, USA). Plasmid pBR322 DNA was obtained from New England Biolabs (Beijing, China). ECL Plus reagent and Polyvinylidene difluoride (PVDF) membrane were products of Bio-Rad Laboratories, Inc. (Hercules, CA, USA). The primary antibodies against γ-H2AX (sc-101696) and anti-mouse (sc-2489) secondary antibody were obtained from Santa Cruz (Dallas, TX, USA). The primary antibodies against β-actin (A5441) were purchased from Sigma. The primary antibodies against cleaved caspase 3 (9662), cleaved caspase 8 (8592), PARP (9542) and anti-rabbit (7074) secondary antibodies were products of Cell Signal Technology, Inc. (Danvers, MA, USA). The primary antibody against thrombospondin-1 (TSP-1) (ab85762) and ceruloplasmin (ab48614) were products of Abcam (Cambridge, UK). Other chemicals were of the highest grade available.

### 2.2. ROS Detection

To detect ROS levels, chemicals were incubated in 0.05 M, pH 7.4 phosphate buffer solution (PBS) with 50 μM DCFH-DA as a fluorescence probe at 37 °C. Fluorescence intensity was measured using a plate reader (Molecular Devices SpectraMax M2*^e^*, Sunnyvale, CA, USA) at an excitation wavelength of 488 nm and an emission wavelength of 525 nm.

### 2.3. Reduction of Cu(II) to Cu(I) by Melatonin

The selective copper sequestering agent BCS was employed to detect Cu(I) by recording the spectra between 350–550 nm [30]. Reaction mixture contained 50 μM Cu(II) and 0.3 mM BCS in 0.05 M PBS (pH 7.4) and indicated concentrations of melatonin, and was incubated at 37 °C for 30 min.

### 2.4. Determination of Hydrogen Peroxide

Polyphenols were incubated at 37 °C in 200 μL PBS (0.02 M, pH 8.0), after which the concentration of hydrogen peroxide was determined according to the protocol provided by the manufacturer (Beyotime Biotechnology, Shanghai, China).

### 2.5. Hydroxyl Radical Detection

Chemicals were incubated in 0.05 M PBS (pH 7.4) with 3 mM 3-CCA as a fluorescence probe at 37 °C [31]. The excitation and emission wavelengths were 388 nm and 446 nm, respectively.

### 2.6. Detection of DNA Damage

DNA cleavage was evaluated by the conversion of supercoiled pBR322 plasmid DNA to open circular and linear DNA forms. Chemicals were mixed in 10 mM Tris-HCl buffer (pH 8.0) and incubated at 37 °C. The DNA samples were loaded by 6 × DNA loading buffer in 1% agarose gel containing Tris acetate-EDTA buffer (40 mM Tris-acetate and 1 mM EDTA, pH 8.0) and 1×Gene Green Nucleic Acid Dye (Tiangen Biotech, Beijing, China), and electrophoresed in a horizontal slab gel apparatus in 1 × TAE gel buffer at 95 V for 1 h. The gel was then photographed under a UV illuminator.

### 2.7. Animals and Treatments

Healthy male Kunming mice (20~22 g, 6~8 weeks) and their diet were purchased from Shanghai SLAC Laboratory Animal Co., Ltd. (Shanghai, China). The mice were housed in plastic cages with controlled temperature (25 ± 1 °C), humidity (50 ± 10%), and 12 h light/dark cycles. The mice were allowed access to food and water ad libitum. Mice were randomly divided into 3 groups (n = 6/group) and were pretreated with saline or melatonin (50 mg/kg) via intraperitoneal (i.p.) injection once daily two times. On the 3rd day, the mice were i.p. injected with saline as control or EGCG (35 mg/kg) plus DEDTC (500 mg/kg) as liver injury model; the mice that received melatonin pretreatment were i.p. injected with EGCG (35 mg/kg) plus DEDTC (500 mg/kg) followed by the 3rd i.p. injection of melatonin (50 mg/kg). All mice were sacrificed by cervical dislocation 24 h later. Peripheral blood from the ophthalmic veins was collected and serum was obtained via centrifugation at 9000× *g* at 4 °C for 10 min. Hepatic tissues were excised and rinsed in ice-cold saline. The serum and liver were then stored at −80 °C before assay. All animals were humanely treated in accordance with the Guidance for the Care and Use of Laboratory Animals of the Ministry of Science and Technology of the People’s Republic of China (2006-398). All animal protocols were approved by the ethics committee of Anhui Agricultural University. All efforts were made to reduce the number of animals used and to minimize their suffering.

### 2.8. Enzymatic Activity Assays

Serum alanine aminotransferase (ALT), aspartate aminotransferase (AST) and ceruloplasmin activities were determined using kits purchased from Jiancheng Bioengineering Institute (Nanjing, China). Liver homogenate was prepared in ice-cold 150 mM PBS (pH 7.2) containing 1 mM EDTA-Na_2_ and centrifuged at 15,000× *g* and 4 °C for 15 min. Serum or hepatic SOD activity was measured using the system of xanthine/xanthine oxidase and nitrobluetetrazolium [32]. One unit of SOD activity was defined as the amount of protein that inhibits the rate of nitrobluetetrazolium reduction by 50%; data of liver or serum were expressed as U/mg protein or U/mL, respectively. Hepatic catalase activity was measured on the basis of its ability to decompose hydrogen peroxide, and the activity unit was defined as nmol hydrogen peroxide consumed/min, data were expressed as U/mg protein [24]. Protein concentrations of the liver homogenates were determined by the Bradford dye-binding assay with bovine serum albumin as a standard.

### 2.9. Copper Measurement

Hepatic tissue (0.5 g) was mixed with 5 mL digestive solution containing 3.75 mL nitric acid and 1.25 mL perchloric acid at 295 °C for several minutes until the appearance of much white smoke and a clear solution. Copper content of the resultant solution was measured using an inductively coupled plasma mass spectrometry with an octopole based collision/reaction cell (Agilent 7700 Series, Waldbronn, Germany). Copper sulfate was used as a standard.

### 2.10. Western Blotting

The concentrations of total protein extracted with the Radio-Immune Precipitation Assay (RIPA) reagent were determined by the BCA protein assay kit (Beyotime Biotechnology, Shanghai, China). Equal amounts of protein were boiled at 95 °C for 10 min in loading buffer and then subjected to SDS-PAGE separation using 10% polyacrylamide gels. After proteins in the gel were electro-transferred to a PVDF membrane, the membrane was blocked with 5% non-fat dried milk in Tris-buffered saline with 0.05% Tween 20 (TBS-T) for 2 h at room temperature. The blot was incubated with specific primary antibodies diluted in TBS-T by 2000 to 5000 folds overnight at 4 °C, and then incubated with secondary antibody diluted in TBS-T by 2500- or 5000-fold for 1 h at room temperature after washing four times with TBS-T. Detection was performed using the ChemiDoc XRS+ detection system (ECL, Bio-Rad, Hercules, CA, USA), and the immunoblots were analyzed with the Quantity One^®^ Image Analyzer software program (Bio-Rad).

### 2.11. RNA Isolation, Reverse Transcriptase Polymerase Chain Reaction (RT-PCR) and qPCR

Total RNA was isolated using TRIzol reagent according to the manufacturer’s protocol (Takara Biotechnology, Kusatsu, Shiga, Japan). RNA samples having a ratio of A_260nm_:A_280nm_ more than 1.8 were used for RT-PCR. Reverse transcription was carried out with 50 ng of total RNA using the reverse transcriptase under the conditions described in the kit manual (RT-for-PCR kit, Takara Biotechnology) in a total volume of 20 μL. Real-time PCR was performed with a CFX System (Bio-Rad) according to the manufacturer’s instructions (Takara Biotechnology). The mRNA level was normalized with reference to the amount of housekeeping gene transcripts *β-actin*. Primers used for PCR reactions were designed using available gene sequences as shown below.

*Thbs1*
Sense: CCCCTACAACCACAACCCTGAC
Antisense: ACTGATCTCCAACCCCATCCAT
*p21*
Sense: GACTTCTCCCATTTCTTAGTAGCAG
Antisense: TGACACCCACGGTATTCAACAC
*Bax*
Sense: TGGAGATGAACTGGACAGCAATAT
Antisense: GCAAAGTAGAAGAGGGCAACCAC
*β-actin*
Sense: GCTGAGAGGGAAATCGTGCGT
Antisense: ACCGCTCGTTGCCAATAGTGA

### 2.12. Statistical Analysis

Data are presented as mean ± SEM (standard error of the mean). The differences between groups were examined by one-way ANOVA *post hoc* Tukey’s or Dunnett’s multiple comparison test upon the *P* value of Bartlett’s test for equal variances using GraphPad software (Prism version 5, San Diego, CA, USA). A *P* value of less than 0.05 was considered statistically significant.

## 3. Results

### 3.1. Melatonin, Copper and Quercetin Synergistically Produce ROS

**Figure 1:**
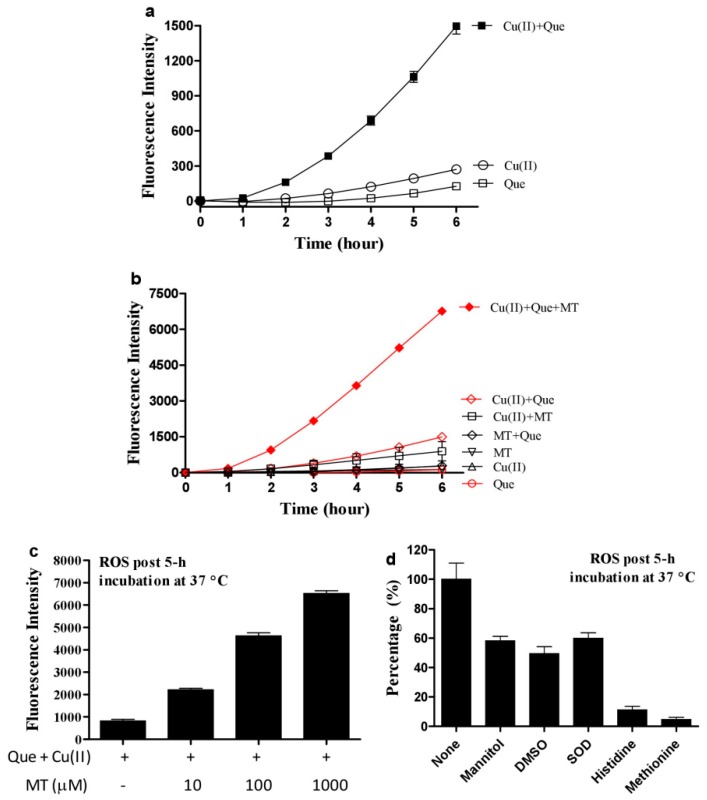
Melatonin enhances ROS production from quercetin and copper. (**a**) ROS production by Cu(II) (1 µM) plus Que (10 µM). (**b**) Melatonin (MT, 100 µM) markedly enhanced ROS formation by Cu(II) (1 µM) plus quercetin (Que, 10 µM). (**c**) Dose-dependent effect of melatonin on ROS production in the redox system of Cu(II) (1 µM) and Que (10 µM). (**d**) Inhibition of ROS formation in the system of 1 µM Cu(II)/10 µM Que/100 µM MT by Mannitol, 100 mM; DMSO, 5%; SOD, 10 U/mL; Histidine, 10 µM; Methionine, 1 mM. Chemicals were mixed in 0.05 M PBS (pH 7.4) and incubated at 37 °C for indicated time. Data are presented as the mean of two replicates; the error bar represents the range.

**Figure 2:**
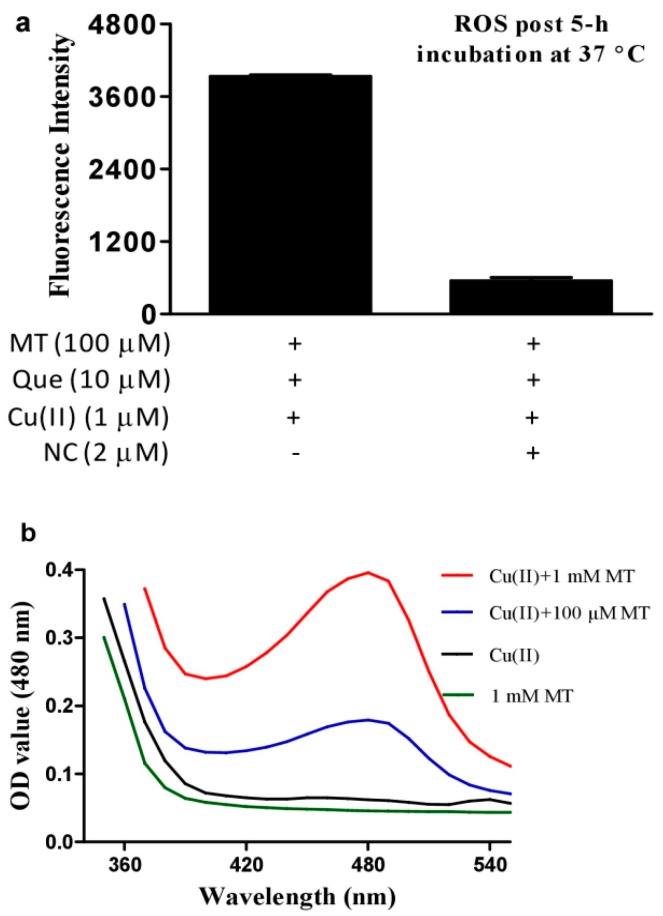
Role of copper (I) for synergistic production of ROS and the influence of melatonin on copper (I) formation. (**a**) Effect of neocuproine (NC) on ROS production in the system of Cu(II), quercetin (Que) and melatonin (MT). Data are presented as the mean of two replicates. (**b**) Detection of MT-induced Cu(I) production by BCS. Reaction mixture contained 50 μM Cu(II) and 0.3 mM BCS in 0.05 M PBS (pH 7.4) and indicated concentrations of MT, and was incubated at 37 °C for 30 min.

Copper ions promote Que autoxidation to generate ROS [33]. This property was recapitulated in our experimental system (Figure 1a). However, melatonin, a well-recognized ROS scavenging agent, dramatically enhanced ROS produced by the combination of Que and Cu(II) (Figure 1b) in a dose-dependent manner (Figure 1c). Methionine and histidine (two well-recognized singlet oxygen scavengers) strongly decreased ROS, whereas sufficient mannitol and DMSO (two typical hydroxyl radical scavengers) or SOD (a superoxide anion scavenger) at an optimal dose selected from a preliminary experiment showed a less inhibitory effect on the ROS signal as compared to the singlet oxygen scavengers (Figure 1d); this suggested that singlet oxygen accounts for the predominant ROS in the system of melatonin, copper and Que. Neocuproine, a Cu(I) sequestering agent [26], substantially reduced the pro-oxidant action of melatonin (Figure 2a), suggesting that melatonin reduced Cu(II) to Cu(I) and drove the production of ROS. To substantiate this speculation, the formation of Cu(I) from Cu(II) in the presence of melatonin was analyzed through Cu(I)-BCS complex with a maximum absorption at 480 nm. As is evident from Figure 2b, in the presence of BCS, melatonin or Cu(II) alone did not lead to any changes in spectra; however, when melatonin was incubated with Cu(II), melatonin dose-dependently increased absorption at 480 nm.

### 3.2. Hydrogen Peroxide Plays an Important Role for Cooperative ROS Production by Melatonin, Copper and Polyphenols

**Figure 3:**
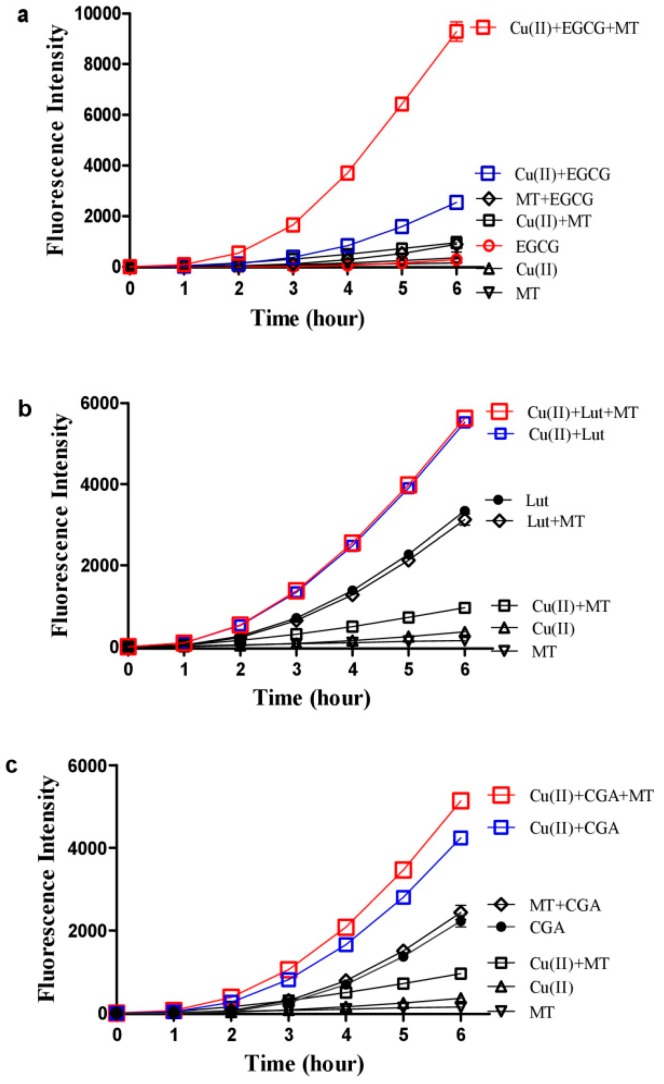
Influence of melatonin on ROS production from copper and other polyphenols. (**a**) EGCG. (**b**) Luteolin (Lut). (**c**) Chlorogenic acid (CGA). In 0.05 M PBS (pH 7.4), the examined polyphenol was 10 µM, the Cu(II) was 1 µM and the melatonin (MT) was 100 µM. The reaction mixtures were incubated at 37 °C. Data are presented as the mean of two replicates; the error bar represents the range. In most data points, the range was smaller than the symbol.

**Figure 4:**
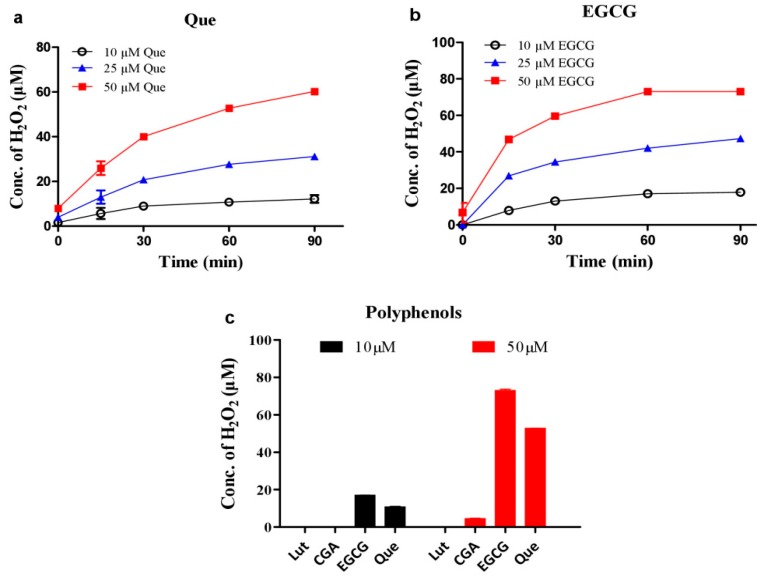
Hydrogen peroxide levels produced by dietary polyphenols. (**a**) quercetin (Que). (**b**) EGCG. (**c**) Comparison of different polyphenols (Lut: luteolin; CGA: chlorogenic acid). Chemicals were mixed in 0.02 M PBS (pH 8.0) and incubated at 37 °C for indicated time in a and b or 1 h in c. Data are presented as the mean of two replicates; the error bar represents the range. In most data points, the range was smaller than the symbol.

**Figure 5:**
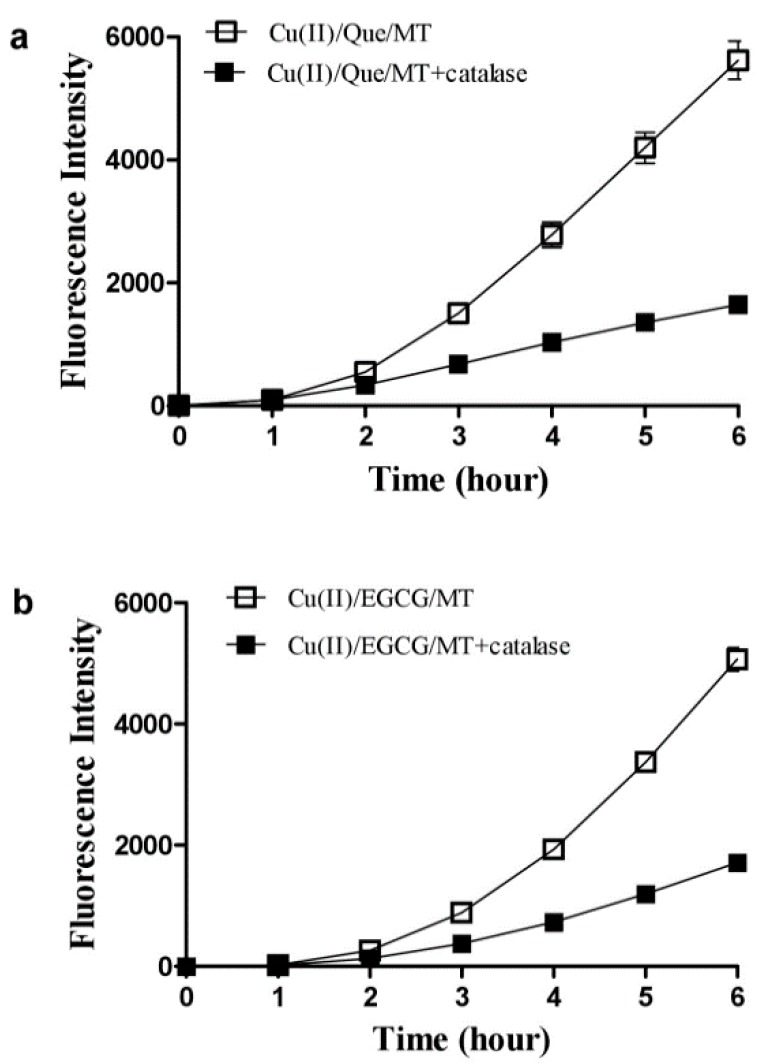
Catalase diminishes melatonin-enhanced ROS production from copper and quercetin or EGCG. (**a**) With quercetin (Que). (**b**) With EGCG. The reaction mixture contained 1 µM Cu(II), 100 µM melatonin (MT), 10 µM EGCG or Que in 0.05 M PBS (pH 7.4) and was incubated at 37 °C. Catalase in the reaction mixture was 5 U/mL. Data are presented as the mean of two replicates; the error bar represents the range; otherwise the range was smaller than the symbol.

In addition to Que, we also investigated whether melatonin stimulates ROS production from copper and other dietary polyphenols. Melatonin enhanced ROS production in a combination of copper and EGCG (Figure 3a), but did not stimulate ROS production from the combination of copper and luteolin or chlorogenic acid (CGA) (Figure 3b,c). Both Que and EGCG produced hydrogen peroxide in time-dependent and dose-associated fashions (Figure 4a,b), whereas luteolin or chlorogenic acid were either less or completely ineffective (Figure 4c). The different effects of these polyphenols appear to correlate with their capacity for producing hydrogen peroxide. Indeed, hydrogen peroxide was an important mediator for the synergistic property due to the ROS induced by melatonin/copper/Que, or EGCG was substantially inhibited by catalase (Figure 5).

### 3.3. DNA Inhibits Melatonin’s Action to Reduce Cu(II) into Cu(I)

**Figure 6:**
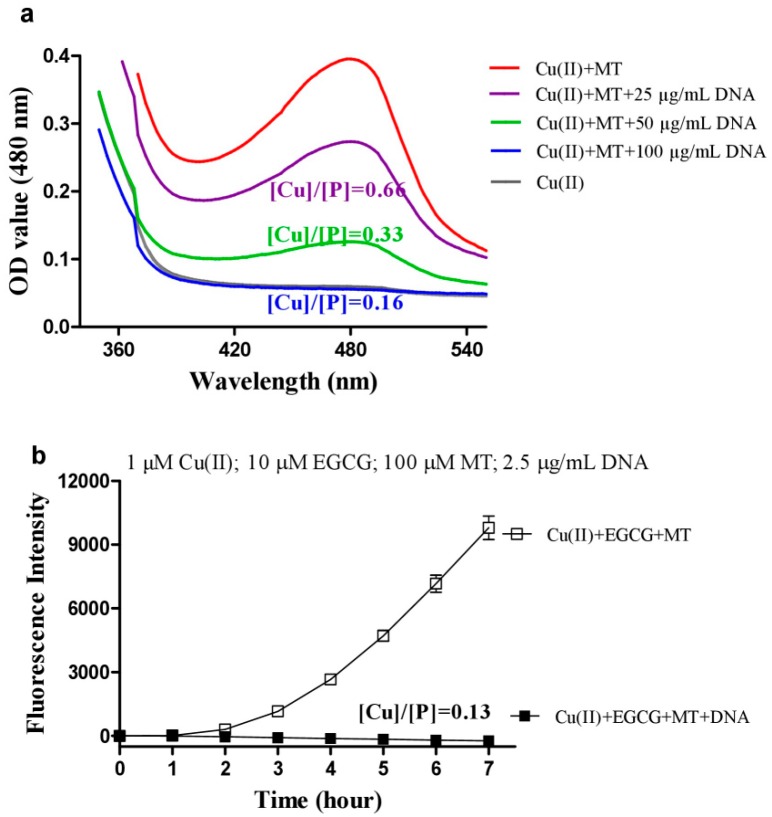
Influence of DNA on melatonin’s actions. (**a**) Detection of melatonin induced Cu(I) production in the presence of DNA. Reaction mixture contained 0.3 mM BCS, 50 μM Cu(II), 1 mM melatonin (MT), and indicated concentrations of DNA in 0.05 M PBS (pH 7.4), and was incubated at 37 °C for 30 min. (**b**) ROS levels. 1 μg/mL (DNA concentration) = 3 μM DNA phosphates (P). [Cu]/[P] is the ratio of the molar concentration of metal ions and DNA phosphates. Chemicals as indicated were mixed in 0.05 M PBS (pH 7.4). Data are presented as the mean of two replicates; the error bar represents the range. In most data points, the range was smaller than the symbol.

Copper ions are mainly resident on DNA in-vivo, the above synergistic ROS production of melatonin, copper and Que or EGCG would mainly occur in an environment with abundant DNA such as the nucleus and mitochondria; we thus examined the influence of DNA on melatonin-caused copper reduction, as well as the synergistic ROS production. As shown in Figure 6a, DNA was effective in inhibiting melatonin’s action in reducing Cu(II) to Cu(I) in a dose-dependent manner, and fully prevented the reduction at high concentration with a molar ratio of [Cu]/[P] being less than 0.2, which is a useful parameter indicating that copper ions bind non-specifically to phosphate groups of DNA [34]. As a result, the synergistic ROS production by copper, EGCG, and melatonin was fully inhibited by DNA at a low [Cu]/[P] molar ratio of 0.13 (Figure 6b).

### 3.4. Melatonin Inhibits Hydroxyl Radical Formed by High Concentrations of Copper/Quercetin or EGCG

**Figure 7:**
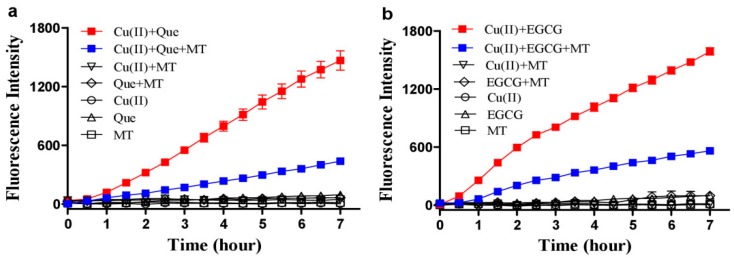
Melatonin inhibits hydroxyl radical formation by copper and quercetin or EGCG. (**a**) Melatonin inhibited the synergistic production of •OH by quercetin (Que) and Cu(II). (**b**) Melatonin inhibited the synergistic production of •OH by EGCG and Cu(II). The concentrations used: 10 μM Cu(II), 100 μM Que or EGCG, and 1 mM melatonin (MT). •OH was measured by 3-CCA (3 mM) fluorescent method. Reactions were conducted in 0.05 M PBS (pH 7.4) at 37 °C. Data are presented as the mean of two replicates; the error bar represents the range. In most data points, the range was smaller than the symbol.

In a redox system of 10 μM copper and 100 μM Que or EGCG (both were 10 times higher than those used in Figure 1, Figure 2, Figure 3, Figure 4 and Figure 5), hydroxyl radical formation was detected by 3-CCA fluorescent probe (Figure 7a,b). Melatonin (1 mM) effectively inhibited the hydroxyl radical formation (Figure 7a,b), in contrast to the above findings that 100 μM melatonin markedly enhanced ROS production in the redox system of 1 μM copper and 10 μM Que.

### 3.5. Melatonin Inhibits DNA Damage Caused by Copper/Quercetin via Chelating Copper

**Figure 8:**
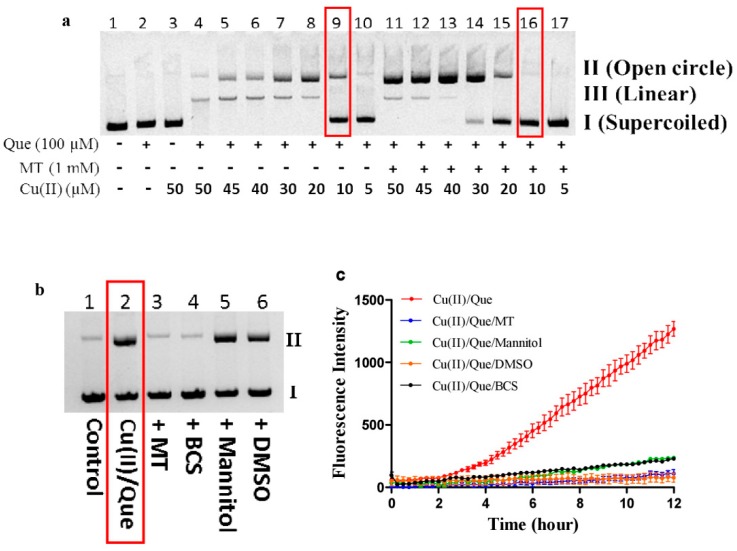
Melatonin prevents DNA from damage induced by copper and quercetin through chelating copper. (**a**) Melatonin (MT) protected against DNA strand breakage induced by quercetin (Que) and various concentrations of Cu(II). (**b**) The role of MT, BCS, mannitol, and DMSO in Que/Cu(II)-induced DNA strand breakage. (**c**) The synergistic production of •OH by Que and Cu(II), and the inhibition by various agents. Concentrations of each tested chemical in b and c were the same, specifically Cu(II), 10 μM; Que 100 μM; MT, 1 mM; Mannitol, 100 mM; DMSO, 5%; BCS, 30 μM. In a and b, all reactions were conducted in Tris-HCl buffer (10 mM, pH 8.0) at 37 °C for 30 min. All reaction mixtures contained 10 μg/mL plasmid DNA. In c, •OH was measured by 3-CCA fluorescent method (3 mM). Reactions were conducted in 0.05 M PBS (pH 7.4) at 37 °C.

Recently, Shao et al. reported that 2, 6-dibromohydroquinone reduced copper in DNA-copper complex to promote peroxidation and the formation of hydroxyl radicals which attack DNA near the copper binding site, leading to the preferential cleavage at guanine, thymine and cytosine residues [31]. Such damage was not prevented by the well-documented hydroxyl radical scavengers, DMSO, or mannitol [31]. Although melatonin prevented hydroxyl radical derived from higher concentrations of copper and polyphenol as shown in Figure 7, whether melatonin protects against DNA damage induced by higher concentrations of copper and polyphenol remains uncertain. The following experiments provided convincing evidence indicating that melatonin is a DNA guardian in the redox system of higher concentrations of copper and Que. As shown in Figure 8a, Cu(II) dose-dependently promoted Que autoxidation to cause DNA fragmentation (form lane 10 to lane 4), and melatonin effectively inhibited the DNA fragmentation (form lane 17 to lane 11). For example, in the presence of 10 μM Cu(II) plus 100 μM Que (lane 9), 1 mM melatonin almost fully prevented the DNA damage (lane 16). Therefore, these conditions were further employed for studying the protective mechanism of melatonin. DMSO or mannitol did not protect against DNA damage (Figure 8b), although they were highly effective in scavenging hydroxyl radicals initiated by the same concentrations of copper and Que in the absence of DNA (Figure 8c). The result is consistent with the concept proposed by Shao et al. that DNA-bound copper triggers Que autoxidation to generate hydroxyl radicals that effectively damage DNA [31]. Under the same condition, BSC, known as a copper chelator or melatonin, readily protected against the damage (Figure 8b), indicating that melatonin acted as a copper chelator.

### 3.6. Melatonin Attenuates the Synergistic Hepatoxicity Induced by EGCG and DEDTC

**Figure 9:**
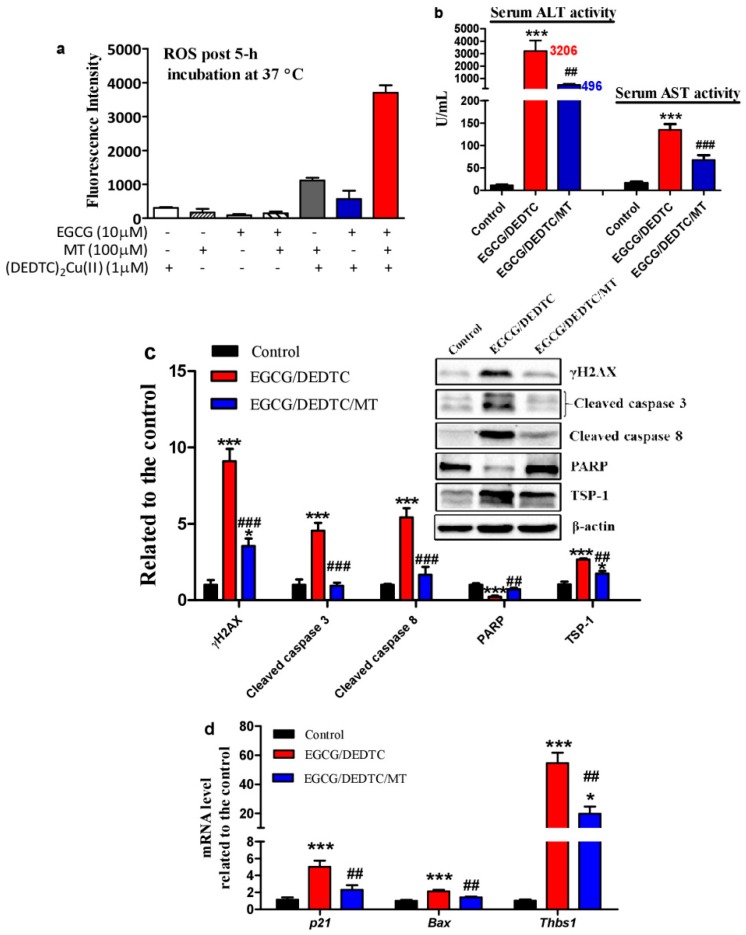
Influences of melatonin on ROS production by EGCG and Cu(DEDTC)_2_ in vitro and oxidative stress induced by EGCG and DEDTC in mice. In-vitro *experiment*: (**a**) Melatonin (MT) enhanced ROS formation of (DEDTC)_2_Cu(II) and EGCG. Chemicals were mixed in 0.05 M PBS (pH 7.4) and incubated at 37 °C. In-vivo *experiment*: Mice were treated with saline as control, EGCG/DEDTC as model of liver injury, and EGCG/DEDTC/MT (drug regimens are described in Materials and Methods). (**b**) Serum ALT and AST activities. (**c**) Hepatic proteins associated with DNA damage, apoptosis and prosurvival effects of nitric oxide. (**d**) p53-associated genes. Data are presented as mean ± SEM (n = 6). Compared to the control, * P < 0.05 and *** P < 0.001. Compared to the DEDTC/EGCG, ## *P* < 0.01 and ### *P* < 0.001. The serum ALT, AST activity (**b**) and *Thbs 1* mRNA level (**d**) were analyzed by one-way ANOVA *post hoc* Dunnett test. The other data (**c**,**d**) were analyzed by one-way ANOVA *post hoc* Tukey’s test.

**Figure 10:**
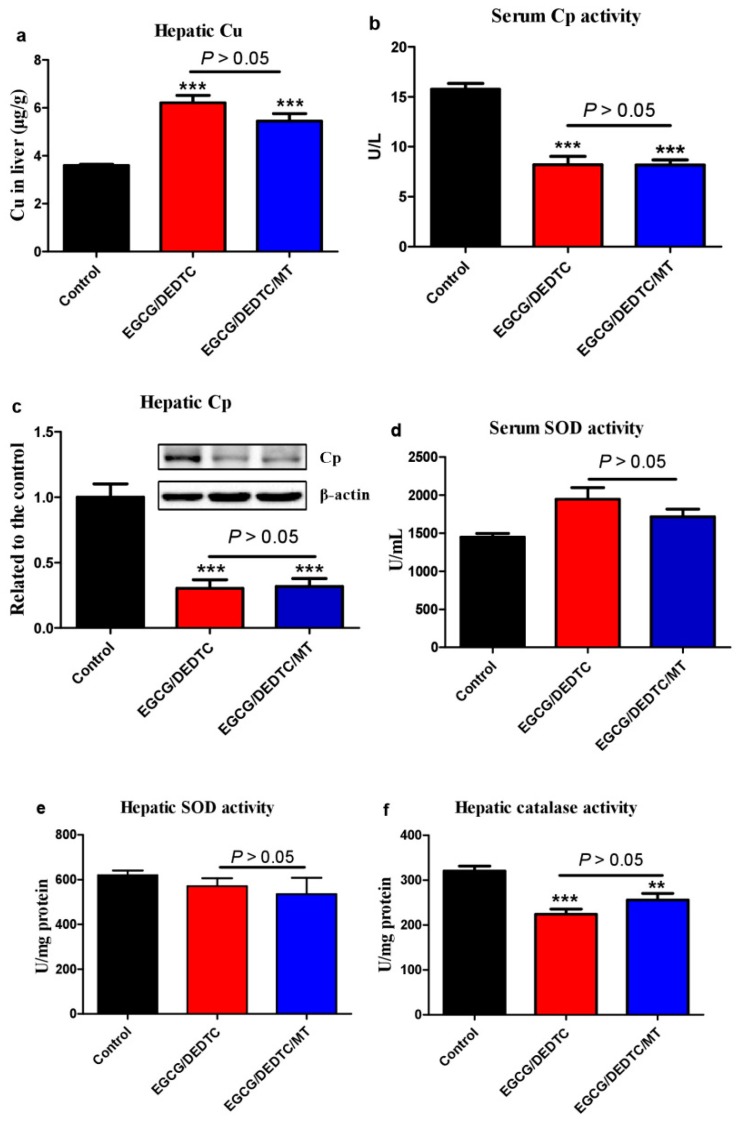
Copper status and antioxidant enzymes responsible for controlling EGCG oxidation in mice. Experimental conditions were the same as Figure 9. (**a**) Hepatic copper levels. (**b**) Serum ceruloplasmin (Cp) activity. (**c**) Hepatic Cp levels. (**d**) Serum SOD activity. (**e**) Hepatic SOD activity. (**f**) Hepatic catalase activity. Data are presented as mean ± SEM (n = 6). Compared to the control, ** P < 0.01 and *** P < 0.001. The hepatic Cu level (**a**) was analyzed by one-way ANOVA *post hoc* Dunnett test and the other data (**b**–**f**) were analyzed by one-way ANOVA *post hoc* Tukey’s test.

Dithiocarbamates can act as a copper ionophore to increase intracellular redox-active copper [35]. It has been reported that the co-administration of EGCG and DEDTC—a metabolite of disulfiram (a drug for alcohol aversion therapy), both at tolerable levels, triggered severe liver injury and lethality in mice [25]. Cu(DEDTC)_2_ complex formation following administration of disulfiram was detected recently in vivo [36]. Herein, we found that melatonin greatly increased ROS levels once it was added into the redox system of Cu(DEDTC)_2_ and EGCG (Figure 9a). Based on these results, we investigated whether melatonin further increases the synergistic hepatotoxicity evoked by the co-administration of EGCG and DEDTC in mice. It has been demonstrated that mice tolerate a single i.p. injection of 35 mg/kg EGCG or 500 mg/kg DEDTC [25]. The co-administration, however, resulted in severe liver injury as evidenced by dramatic elevation of serum ALT and AST activities (Figure 9b). Associated with the liver injury, there were: (1) hepatic DNA damage as suggested by the increase of hepatic γH2AX (Figure 9c), (2) hepatocellular apoptosis as indicated by the increase of hepatic cleaved caspase 3 and 8 as well as the reduction of hepatic PARP (Figure 9c), (3) blockade of prosurvival effects of nitric oxide as suggested by increased TSP-1 and *Thbs1* (Figure 9c,d), [37,38] and (4) hepatic p53 activation as reflected in up-regulation of *p21, Bax* and *Thbs1* mRNA (Figure 9d). Interestingly, these deleterious alterations were significantly alleviated by melatonin (Figure 9b–d). On whether the in-vivo protective effect of melatonin is associated with its influence on copper homeostasis, we found that melatonin did not significantly prevent the EGCG and DEDTC co-administration-induced hepatic copper elevation (Figure 10a) and the reduction of serum ceruloplasmin activity or hepatic ceruloplasmin levels (Figure 10b,c). In the copper-enhanced EGCG toxicity model, the protective effect of melatonin did not involve its influence on SOD and catalase (Figure 10d–f). These enzymes are known to prevent the oxidation and cytotoxicity of EGCG [39,40,41]. These lines of evidence suggest that melatonin acts as a direct antioxidant in vivo to attenuate the synergistic hepatoxicity of EGCG and DEDTC.

## 4. Discussion

At first glance, it appears paradoxically that melatonin promotes low concentrations of copper and polyphenol to produce ROS. We thus investigated the underlying mechanism. The synergistic production ROS was closely associated with copper reduction because BCS, a copper (I)-specific sequestering agent, abolished the synergistic effect. Consistent with the findings that the precursor of melatonin (serotonin) or a metabolite of melatonin (6-hydroxymelatonin) can reduce Cu(II) to Cu(I) [8,9,10], we found that melatonin lowered Cu(II) to Cu(I) dose-dependently. Thus, the capacity of melatonin to promote copper reduction is involved in the synergistic production of ROS. In addition, hydrogen peroxide is a crucial mediator for the synergistic effect because (1) the synergistic effect was only observed from those polyphenols with a strong ability of producing hydrogen peroxide such as Que and EGCG, but was not seen using those polyphenols without a strong ability to form hydrogen peroxide such as luteolin and chlorogenic acid; (2) catalase efficiently eliminated the synergistic effect; and (3) an early study showed that melatonin dramatically enhanced ROS production in a Fenton-like reaction system of hydrogen peroxide and copper [7]. In this earlier study, singlet oxygen was identified as a predominant ROS [7]; herein, we also found that the major ROS in the synergistic system was singlet oxygen. Overall, polyphenol-derived hydrogen peroxide and melatonin-facilitated copper reduction are two important elements underlying the synergistic production of ROS.

The potential influence of such a synergistic effect of ROS production on DNA constitutes a concern because nuclear copper is mainly resident on DNA and copper/phenolic compounds cause DNA damage [29]. Thus, we investigated the effects of melatonin on copper and the synergistic effect between copper and polyphenols in the presence of DNA. We found that DNA dose-dependently inhibited the reduction of copper by melatonin; as a result, DNA inhibited the synergistic action. DNA can bind copper and the binding manner is dependent on molar ratio of [Cu]/[P] [34]. Below 0.2, copper ions bind non-specifically to phosphate groups. In a range of 0.2–0.4, some copper ions begin to bind the N7 of guanine and the N3 of cytosine. In a range of 0.4–0.7, guanine-cytosine stacking of copper becomes appreciably lower [34]. The molar ratios of [Cu]/[P] used in the present study were 0.13–0.16, 0.33, and 0.66, which fall into the three typical binding capabilities of copper and DNA. Based on the results of the effects of DNA on copper reduction and the synergistic production of ROS (Figure 6), it may be concluded that when copper ions bind to the phosphate groups of DNA, copper is no longer be reduced by melatonin. Thus, ROS formation was not observed (Figure 6) and DNA damage is less likely to occur. This conclusion is also consistent with our in-vivo experiment, demonstrating that melatonin in turn protected against hepatic DNA damage induced by EGCG and DEDTC, which increases liver copper levels (Figure 9). Such a protective action of melatonin in vivo is probably due to its inhibitory effect on hydroxyl radical production from EGCG and copper, which was observed in vitro (Figure 7), without induction of hepatic SOD and catalase (Figure 10). These enzymes are known to suppress EGCG oxidation [39,40,41]. In addition, the present in-vivo experiment reinforces the idea of using melatonin to alleviate potential adverse effects of EGCG. EGCG has shown many beneficial health effects; however, high-dose EGCG is apt to trigger hepatotoxicity [19,20]. We have previously demonstrated that melatonin ameliorated EGCG-induced hepatotoxicity in mice [42] and enhanced anticancer activities of EGCG in vitro [43]. We recently showed that EGCG and DEDTC, both at a safe dose, synergistically induced hepatotoxicity in mice because DEDTC-increased hepatic copper promoted EGCG oxidation [25]. The present in-vivo experiment provides evidence indicating that melatonin is also highly effective in attenuating the synergistic hepatotoxicity of EGCG and DEDTC.

Melatonin with conditional pro-oxidant actions [12,13] is a powerful scavenger of hydroxyl radical [44,45]. Physicochemical analysis using the density functional theory suggests that melatonin is a copper chelating agent [5]. Currently, the mechanism by which melatonin protects against copper-mediated DNA damage is postulated to be associated with melatonin’s capacity of chelating copper and/or scavenging hydroxyl radical [46,47]. However, the fundamental action responsible for the protective effect remains unclear. Shao et al. recently demonstrated that the Cu(I) in DNA-copper complexes reacted with hydrogen peroxide produced by the redox reaction of 2,6-dibromohydroquinone and Cu(II) to produce highly reactive hydroxyl radicals near the binding site of copper and DNA, leading to immediate and site-specific DNA damage, which could not be salvaged by sufficient amounts of DMSO or mannitol, two well-recognized hydroxyl radical scavengers [31]. In the present study, we showed that copper/Que-induced DNA damage also was unable to be prevented by excessive amounts of DMSO and mannitol, although they were highly effective in scavenging hydroxyl radical initiated by copper/Que in the absence of DNA. This suggests that DNA-bound copper readily reacted with Que-derived hydrogen peroxide to produce hydroxyl radical which in situ and site-specifically damages DNA. However, the copper chelator BSC or melatonin effectively protected against the DNA damage. Thus, concerning copper-mediated DNA damage, melatonin acts as a copper chelator to prevent hydroxyl radical formation, rather than as a scavenger of hydroxyl radical, to prevent DNA from damage. The present study reveals that the functions of melatonin in scavenging hydroxyl radical and chelating copper for protection of DNA are not redundant; melatonin with the function of chelating copper and scavenging hydroxyl radical is a more reliable DNA guardian than antioxidants that only have a single function of scavenging hydroxyl radical.

## 5. Conclusions

**Figure 11:**
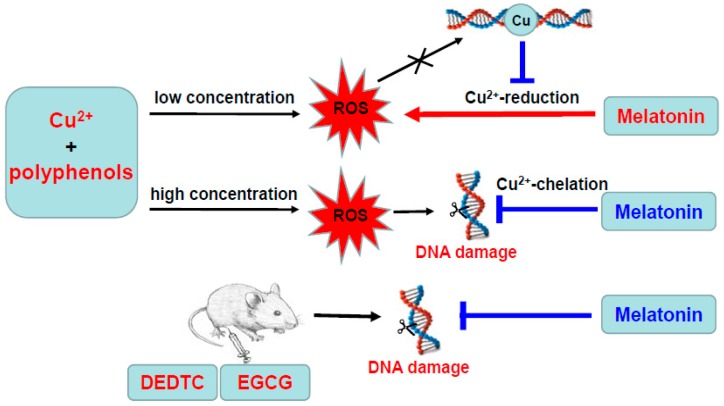
Summary diagram of the present study.

As is outlined in Figure 11, the present study describes the pro-oxidant and antioxidant actions of melatonin in a redox system of copper and polyphenols. Melatonin promotes ROS production in a system containing low concentrations of copper and polyphenol via reducing copper; however, the presence of DNA prevents melatonin from reducing copper and inhibits ROS production. With high concentrations of copper and polyphenol, melatonin decreases hydroxyl radical formation and DNA damage by chelating copper to prevent the formation of hydroxyl radical. Interestingly, melatonin administration protects against copper/polyphenol evoked DNA damage in vivo. The results elucidate the actions of melatonin under different redox conditions and suggest that melatonin may prevent against copper-sensitized polyphenol toxicity in the liver.
